# Expression responses of *XTH* genes in tomato and potato to environmental mechanical forces: focus on behavior in response to rainfall, wind and touch

**DOI:** 10.1080/15592324.2024.2360296

**Published:** 2024-05-29

**Authors:** Norbert Hidvégi, Judit Dobránszki, Bianka Tóth, Andrea Gulyás

**Affiliations:** Centre for Agricultural Genomics and Biotechnology, Faculty of the Agricultural and Food Science and Environmental Management, University of Debrecen, Nyíregyháza, Hungary

**Keywords:** Abiotic stress, mRNA expression level, mechano-stimulus, transcriptome, xyloglucan endotransglucosylase/hydrolase

## Abstract

Rainfall, wind and touch, as mechanical forces, were mimicked on 6-week-old soil-grown tomato and potato under controlled conditions. Expression level changes of xyloglucan endotransglucosylase/hydrolase genes (*XTH*s) of tomato (*Solanum lycopersicum* L. cv. Micro Tom; *SlXTH*s) and potato (*Solanum tuberosum* L. cv. Desirée; *StXTH*s) were analyzed in response to these mechanical forces. Transcription intensity of every *SlXTH*s of tomato was altered in response to rainfall, while the expression intensity of 72% and 64% of *SlXTH*s was modified by wind and touch, respectively. Ninety-one percent of *StXTH*s (32 out of 35) in potato responded to the rainfall, while 49% and 66% of the *StXTH*s were responsive to the wind and touch treatments, respectively. As previously demonstrated, all *StXTH*s were responsive to ultrasound treatment, and all were sensitive to one or more of the environmental mechanical factors examined in the current study. To our best knowledge, this is the first study to demonstrate that these ubiquitous mechanical environmental cues, such as rainfall, wind and touch, influence the transcription of most *XTH*s examined in both species.

## 1. Introduction

Plants are constantly exposed to the effects and stresses of their natural environment. As being sessile organisms with limited spatial mobility, they cannot escape but have had to develop advanced strategies to cope with and adapt to them.^1–4^

While growing and developing, plants adapt continuously to environmental changes in order to survive, better utilize the resources from nature and mitigate the environmental stresses. External environment-borne mechanical forces have been neglected for a long time, but their importance has been rediscovered in recent decades. Perceiving and responding to them, their evolutionary importance, moreover, their role in the plant growth, development, thigmomorphogenesis and stress mitigation has now begun intensively studied.^2,3,5–10^ The most obvious and known mechanical forces and perturbations affecting the everyday life of plants include wind pressure, rainfall, water flow, injuries caused by animals and humans, sound, ultrasound and touch.^2,6–8,10,11^

After applying wind, touch, rain, wounding and dark treatments to *Arabidopsis thaliana* ecotype Colombia plants, five touch-inducible genes (*TCH1–5*) were identified by Braam and Davis.^12^ *TCH1* encodes CAM2, a calmodulin, *TCH2–3* encodes calmodulin-like proteins (CML24, CML12), while *TCH4* encodes XTH22, which is a xyloglucan endotransglucosylase/hydrolase. Later it was proved that *TCH4* could be induced also by sound waves (50 Hz, for 30 min) in *Arabidopsis thaliana*.^13^ Afterward, a series of genes sensitive to mechano-stimuli was identified including genes encoding different calmodulins, protein kinases and other proteins (summarized in Lee et al.^14^), among them XTHs (xyloglucan endotransglucosylase/hydrolase), as well.

XTHs play role in the cell growth due to break down and even rejoin crosslinks of hemicellulose polymers in the cell wall, thereby causing loosening, extension and restructuring of the cell wall.^15–18^ Some *XTH* genes were recently proved to be mechano-inducible not only in *Arabidopsis thaliana* Columbia (Col-0)^11,14^ but in cucumber (*Cucumis sativus* L. cv. Borszczagowski)^19^ and potato (*Solanum tuberosum* L. cv. Desirée),^20^ as well. In *Arabidopsis thaliana*, 589 genes (above 2.5% of the total genes of *Arabidopsis*) were proven to be inducible by touch, while 171 genes responded with down-regulation.^14,21^ Studying the 33 *XTH* genes of *Arabidopsis thaliana* (*AtXTH1–33*), the expression level of four *AtXTH*s (*AtXTH17, AtXTH22, AtXTH25* and *AtXTH31*) increased but that of 3 *AtXTH*s decreased after touch stimulus,^14^ while only one *AtXTH* (*AtXTH22*) was up-regulated in response to sound stimulus.^11^ Two cucumber *XTH* genes (*CsXTH1* and *CsXTH3*), which are up-regulated during somatic embryogenesis, were investigated for evaluating the change of their expression intensities in response to mechanical stimuli, such as touch and wounding. After applying mechanical stimuli the promoters of those *XTH*s were activated.^19^ These investigations indicated that the studied *CsXTH*s were regulated not only developmentally but by mechanical stimuli, as well, due to the correlation between the developmental processes and mechanical stresses occur during growth, differentiation and development.^21^ In tomato, 25 *XTH* genes (*SlXTH1–25*) were identified,^22^ but their reply to mechano-stimulus was not examined. With the corresponding 13 sequences, 11 *XTH* genes (*StXTH1*, *StXTH2*, *StXTH3*, *StXTH5*, *StXTH6*, *StXTH7*, *StXTH9*, *StXTH10*, *StXTH12*, *StXTH16* and *StXTH25*), which are putatively homologous to *AtXTH*s and/or *SlXTH*s, were identified. For identification of these 11 *StXTH*s, an *XTH* homology search was used based on Gene Ontology and functional annotation, and they were proven to be responsive to ultrasound in potato either by up- or down-regulation.^20^

In the present experimental study, we have examined the *XTH* genes described in tomato (*SlXTH*)^22^ and the *XTH* genes of potato (*StXTH*) identified earlier by our group and some of them were proven to be responsive to ultrasound.^20^ We have studied if their expression intensities can be changed by different other mechanical stimuli, like wind, rain and touch; moreover, what is the relationship between the responses to the various mechano-stimuli and in each species.

## 2. Materials and methods

### 2.1. Plant material and growth

Two plant species, tomato (*Solanum lycopersicum* L. cv. Micro Tom) and potato (*Solanum tuberosum* L. cv. Desirée) were used in the experiments. The permission was obtained for purchase of tomato seeds and was obtained for collection of potato plants from *in vitro* gene bank of Centre for Agricultural Genomics and Biotechnology. Tomato seeds were each sown into pots of 10 cm in diameter. Potato plants were derived from *in vitro* culture; *in vitro* 4-week-old potato plantlets were planted individually into pots of 10 cm in diameter and acclimated for 2 weeks. The soil in pots consisted of a 1:1 mixture of perlite and forest soil.

Culture of plants was conducted under 16-h-light and 8-h-dark regime, at 22 ± 2°C daytime and 16 ± 2°C night time temperature, with 60% humidity in the culturing room. Plants were grown for 6 weeks before performing mechanical force treatments.

### 2.2. Mechanical force treatments and collection of samples

Rainfall, wind and touch were imitated by performing mechanical force treatments on 6-week-old tomato and potato plants.

*Rainfall*. A flower sprayer bottle (Cortex 2 l HY007–3) completely filled with distilled water was used to simulate the rainfall. Each treated plant was sprayed by two pressures of the spray bottle 3-times one after another, and delivering a total of 20 ml of distilled water onto the top of the plants from a distance of 15 cm.

*Wind*. A closed box with transparent walls was used to mimic wind pressure. A computer fan (Foxconn^Ⓡ^ DC Brushless Fan, Model: PV802512L1SF 2E, DC12 V, 0.20 A) was installed into the top wall of the box. Plants were placed into this box, 10 cm from both the top wall and the computer fan, and they were blown with the airflow from the computer fan for 5 min. Each control plant was placed in a similar box but without a computer fan for 5 min.

*Touch*. Touch stimulation was similar to that described by Johnson et al.^13^ Each shoot was touched lengthwise from above, with both hands, and bent 10-times to the right and left.

Each force treated and control plant was cultured under the same conditions as described above. Sampling was made immediately (at 0 min), at 15 min, 30 min and 24 h after mechanical force treatments. Three-three independent samples were collected from each treatment and their controls, in each time point from both species. Whole plants of both treated and control plants were collected at each time point and immediately frozen in liquid nitrogen. All plant samples were stored and kept at −80°C until RNA expression analysis.

### 2.3. Quantification of XTH gene expression by real-time PCR

Total RNA was isolated using Direct-zol™ (Zymo Research, Irvine, CA, USA) with TRIzol reagent based on the manufacturer’s manual. After total RNA extraction, three quality control methods were applied: 1) microcapillary electrophoresis with Implen n50 spectrophotometer (Implen, Munich, Germany); 2) agarose gel electrophoresis; 3) Agilent Bioanalyzer 2100 system to check the quality and quantity of total RNA. Total RNA of 120 ng was used for first-strand cDNA amplification with FIREScript RT cDNA Synthesis MIX (Solis BioDyne, Tartu, Estonia). The second-strand cDNA synthesis was performed by 5 × HOT FIREPol EvaGreen qPCR Synthesis MIX (Solis BioDyne, Tartu, Estonia) on ABI 7300 real-time PCR system (ThermoFischer Scientific, Waltham, MA, USA). Specific primers for *SlXTH* genes were used from Saladié et al.^22^

The UniPro UGENE v39.0 program^23^ was used to design specific primers for *StXTH*s based on the previous study about the identified homologous *StXTH* genes.^20^ We selected five commonly used reference genes (*EF1α*, *elongination factor-1alpha*; *actin*; *GAPDH*, *glyceraldehyde-3-phosphate dehydrogenase*; *sec3*, *exocyst complex component sec3*) as normalizing genes for RT-qPCR based on the Tang et al.^24^ results on validated reference genes in potato under abiotic stress. To compare the stability of expression intensity among the candidate reference genes, we used several statistic methods: geNorm,^25^ NormFinder,^26^ and BestKeeper^27^ based on the cycle quantification value (Cq). The results were compared from the geNorm, NormFinder, and BestKeeper with the comprehensive ranking platform RefFinder^28^ which based on the geometric mean of the rankings of every single gene calculated by each statistical program. In the RT-qPCR analysis, we used 2^−∆∆Ct^ method to quantify the relative changes in gene expression.^29^ Gene expression logarithmic fold change (log2FC) was calculated for comparing the intensity of *SlXTH* and *StXTH* gene expression between the control and treatment. The Student’s *t* test (Independent-Samples T test) was performed on ∆∆Ct values pairwise, by SPSS for Windows (SPSS^Ⓡ^, version 25.0) and p-value less than 0.05 was considered to be significant. Log2FC values were used to present the results.

The TBtools v1.09852^30^ was used for visualizing the significantly differently expressed *XTH*s on the whole genome of potato and tomato based on the *Solanum tuberosum* SolTub 3.0 (https://plants.ensembl.org/Solanum_tuberosum/Info/Index) and *Solanum lycopersicum* SL3.0 (https://plants.ensembl.org/Solanum_lycopersicum/Info/Index) genome databases.

The study complies with local and national guidelines.

## 3. Results

Replies of 25 *SlXTH*s identified by Saladié et al.^22^ to three mechanical forces were identified. Changes of expression intensities of 35 potato sequences originated from RNAseq data sets,^31,32^ which are putatively homologous to 20 *AtXTH*s and 21 *SlXTH*s, and thereby putatively *StXTH*s,^20^ were studied in response to the rainfall, wind and touch. Specific primers designed for *StXTH*s are presented in Table 1.

**Table 1.** Sequences of primers designed for *StXTH* genes.

Gene	Forward primer sequence (5’-3’)	Reverse primer sequence (5’-3’)
StXTH19	TGGATAAATCCTCAGGAGCTGGA	ACCCCTGCATCTTGATCATCTG
StXTH4–1	GGACCAATCTTCTGGATCTGGC	GGTTTGCCTTCTCTATTGCCCA
StXTH4–2	ACAGTCACTGCTTTCTACTTGTCTT	TCACCTTTCCCTCCCGTGTA
StXTH1	GTTGTCACTGCATTTTACCTGTCAT	GTAGCCCTTGGTTGGGTCAAA
StXTH18	AAACACAGGGGTTGGTGTCAA	ACTCAAATCCGCAGCCAGAAG
StXTH4–3	TGTTCACAGGAGGCAAAGGAG	CGTCGTCCACAAAGATCACAATTT
StXTH7	ACCATCAAGCCGTGTTCTCC	CTAAGCCACCCCTTGTAGCC
StXTH23–1	ACCTTAGACCAAGTATCAGATTGTG	TCTCAATGGCCCTGCCTCTA
StXTH15	ACACAAGCTGAAGTACAAGGTTCA	TGGTCATAAATCCACATCCTGCTT
StXTH16	AGACCATCTTTCTGGTACTGGG	TGACAGTTCCAGCAGAGTCAC
StXTH7	AACACTTCTGTTGATGGCCGTA	TCGGTTGACCCGAAGCTAAATAA
StXTH10–1	CAGCGCGCTCAGATACAAGA	CCTGCTCCTTGAGAAGACAATAAGA
StXTH10–2	CACTGTCACCACATTCTACTTGTCT	TAAGGTTGTCCTGATGAATTTCCCA
StXTH12	ATGCCCAAGGCAAGGGTAAC	GGTGTGTTGTCCACCAAAAATATGA
StXTH9–1	TCTTGGGAAATGTGACGGGTG	TCATCCACCAAGAACACTATGCG
StXTH3–1	TTTAGCCAAGGCAAAGGAGACA	TGTTGTCCACCAAGAAAATGATGTG
StXTH13	CGTGTTGTTGACAGCCTCCC	GACTGAAAGCCTGATCCAGACA
StXTH3–2	ATGCAACTTAAGCTCGTGCG	TGTTGACCCTTGTGATGATAAATAA
StXTH10–3	GTTCTGCAACGGATGTGCCT	GTGATTCCTGCATTGTCCCTACA
StXTH3–3	TTGGAATCCACAACGCATCATATTC	CCTCATTGGTTGGTTCTTCGGAT
StXTH2	GGGAATTTGAGTGGTGACCCA	GGTGTCCCATCTACTGAAAATATGA
StXTH9–3	GGAATCCTCATCGCATAGTGTTCT	TGAAAGGTGCAAGTGCCCAA
StXTH9–2	GGAATCCACATCGCATAGTGTTCT	AGCAGTGAATGGTGCAAGGG
StXTH20	GCACTGTCACTGCTTACTATTTGTC	GGCTCTCCACTCACATTACCAA
StXTH8–1	GGACTGACAGCCACATCATCTTT	CTTGGTGACGTAAGGGGCAT
StXTH8–2	AGCTTTTGATGAAGGCTACTCTCA	GACACAAATCCAGCCCCTGT
StXTH5–1	GCCATCTCTTTGGTGATGGAAAC	AGACTTGAAACCTGAACCTGTGT
StXTH5–2	GTACATTTTTCCGGCGGCTG	TGTTGAAATTACACCCGATCCTGA
StXTH23–2	GGGATGCTTCATCATGGGCT	TTCCGACGAATGGTTGGTACTTAT
StXTH6–2	TGGGACCCCAATGAGATCATATTTT	CTTCCTCCGTTGCCCAAGAT
StXTH6–1	GGGATGCTTCATCATGGGCT	TTCCGACGAATGGTTGGTACTTAT
StXTH14	TGGGGTCCTAATCATCAAAGTGTAG	CCGACTTGAATCCACTTCCTGA
StXTH25	CTCATGTCGAGGTCCAGTGT	AGAGATTAGTCCAGAACCTGAAGA
StXTH23–3	GCCCTGATGTGAGAGGAACC	CTGCATGACACACATAACTGGC

**Figure 1.**
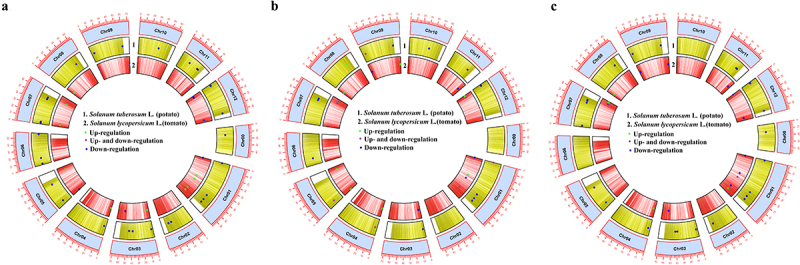
Circular view of the *XTH* gene density on potato and tomato chromosomes (Chr) under rainfall (a), wind (b) and touch (c) treatments based on the TBtools^30^.

*XTH* genes of potato and tomato, respectively, which expression level changed at any of the time points (0 h, 15 min, 30 min or 24 h) examined after mechanical forces applied in this study are presented in Figure 1. Each *XTH*s, which showed altered gene expression in response to rainfall (Figure 1A), wind (Figure 1B), or touch (Figure 1C), was plotted based on its position on the chromosome.

### 3.1. The effect of rainfall treatment on the gene expression of XTHs

**Figure 2.**
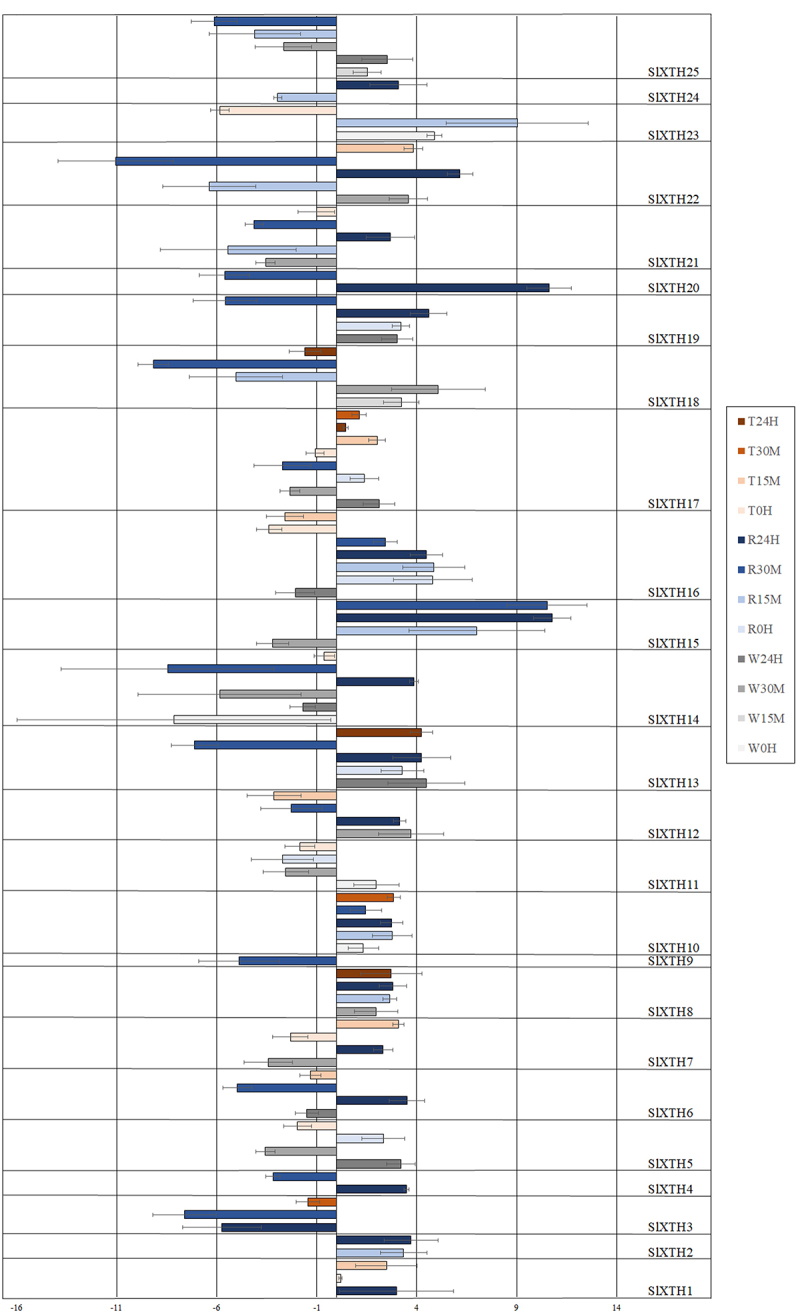
Expression induction and repression of *SlXTH*s of tomato in rainfall - (R), wind - (W), and touch - (T) treated plants at different time point (0 hour, 15 min, 30 min and 24 hours), assessed by real-time PCR (Average of Log2FC values from three independent samples are presented by bars. Standard deviation is represented by error bars).

*Tomato*. All 25 *SlXTH*s responded to the rainfall treatment and significantly altered their expression levels, at least for one of the time points studied (Figures 1A, 2). The expression level of *SlXTH16* showed a significant increase in response to rainfall at all time points. The expression level of *SlXTH5*, and *SlXTH11* changed only immediately after rainfall treatment (at 0 h), but in the opposite way, i.e. expression of *SlXTH5* decreased, but that of *SlXTH11* increased at 0 h. Expression of *SlXTH1* and *SlXTH7* increased significantly in response to the rainfall but only at 24 h. Similarly, an increased gene expression of *SlXTH23* was detected at 15 min. Gene expression of *SlXTH9* was repressed at 30 min. Other *SlXTH*s responded at least two time points to this mechanical cue. *SlXTH2*, *SlXTH8, SlXTH10, SlXTH15* were induced after rainfall treatment, while *SlXTH3, SlXTH18* and *SlXTH25* were repressed. In the case of 8 *SlXTH*s (*SlXTH4, SlXTH6, SlXTH12, SlXTH14, SlXTH20, SlXTH21, SlXTH22, SlXTH24*) the gene expression was repressed at the first time point, while it was increased later. Just the opposite response was observed in the case of *SlXTH17*, i.e. it was induced at 0 h and repressed at 30 min. Gene expression was induced at 0 h and 24 h but repressed at 30 min in *SlXTH13* and *SlXTH19*.

**Figure 3.**
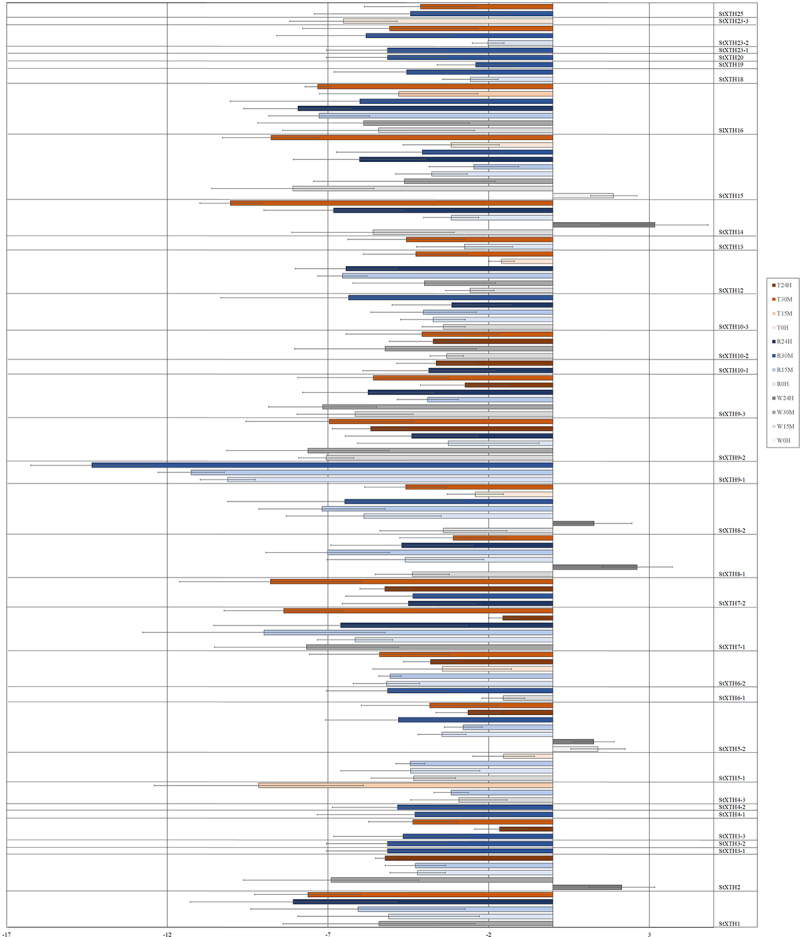
Expression induction and repression of *StXTH*s of potato in rainfall- (R), wind- (W), and touch- (T) treated plants at different time point (0 hour, 15 min, 30 min and 24 hours), assessed by real-time PCR (Average of Log2FC values from three independent samples are presented by bars. Standard deviation is represented by error bars).

*Potato*. The 32 *StXTH*s responded to rainfall treatment by significantly changing the expression level of each (Figure 1; Figure 3). The expression level of *StXTH6–3*, *StXTH10–2* and *StXTH23–3* showed no significant changes. The expression level of other *StXTH*s showed a significant decrease.

### 3.2. The effect of wind treatment on the gene expression of XTHs

*Tomato*. Seven out of 25 *SlXTH*s (*SlXTH1, SlXTH2, SlXTH3, SlXTH4, SlXTH9, SlXTH20, SlXTH24*) were not sensitive to wind treatment (Figures 1B, 2). Two *SlXTH*s (*SLXTH10* and *SLXTH23*) responded to the airflow only at the first time point (0 h) by increased gene expression, while the expression level of four others (*SlXTH6, SlXTH13, SlXTH16* and *SlXTH19*) changed only at the time point of 24 h. The expression of both *SlXTH13* and *SlXTH19* was induced, while that of *SlXTH6* and *SlXTH16* was repressed. *SlXTH8, SlXTH12, SlXTH18* and *SlXTH22* were induced at 30 min, as well as *SlXTH18* at 15 min, while *SlXTH7, SlXTH14, SlXTH15* and *SlXTH21* were repressed at 30 min, as well as *SlXTH14* at 0 h and 24 h. The expression level of *SlXTH5*, *SlXTH11*, *SlXTH17* and *SlXTH25* was either increased or decreased depending on the time point.

*Potato*. The 18 out of 35 *StXTH*s (*StXTH3–1*, *StXTH3–2*, *StXTH3–3*, *StXTH4–1*, *StXTH4–2*, *StXTH6–2*, *StXTH6–3*, *StXTH7–2*, *StXTH9–1*, *StXTH10–1*, *StXTH13*, *StXTH18*, *StXTH19*, *StXTH20*, *StXTH23–1*, *StXTH23–2*, *StXTH23–3* and *StXTH25*) were not sensitive to wind treatment (Figures 1B, 3). The expression level of *StXTH5–2* was induced at time point of 0 h. The expression of *StXTH8–1*, *StXTH8–2* and *StXTH14* was decreased at 15 min but was increased at 24 h. The gene expression level of *StXTH15–2* was increased at time point of 0 h and was decreased at 15 min.

### 3.3. The effect of touch treatment on the gene expression of XTHs

*Tomato*. Nine *SlXTH*s (*SlXTH2, SlXTH4, SlXTH9, SlXTH15, SlXTH19, SlXTH20, SlXTH21, SlXTH24* and *SlXTH25*) were not sensitive to touch treatment (Figures 1C, 2). The expression level of *SlXTH17* altered significantly at each time point after touch treatment; it was repressed immediately after touch treatment but was induced later in each remaining time point. *SlXTH5, SlXTH11*, *SlXTH14* and *SlXTH23* responded immediately after touching (at 0 h); *SlXTH23* was induced, while the three others were repressed. Three other *XTH*s (*SlXTH8, SlXTH13*, and *SlXTH18*) altered their gene expression only at the last time point (at 24 h after touching), *SlXTH8* and *SlXTH13* were induced, while *SlXTH18* was repressed. The expression level of *SlXTH1*, *SlXTH10* and *SlXTH22* increased, while that of *SlXTH3*, *SlXTH6*, *SlXTH12* and *SlXTH16* decreased at one or two time points after the touch treatment. *SlXTH7* was repressed at 0 h but then induced at 15 min.

*Potato*. The 12 out of 35 *StXTH*s (*StXTH3–1*, *StXTH3–2*, *StXTH4–1*, *StXTH4–2*, *StXTH6–1*, *StXTH6–3*, *StXTH9–1*, *StXTH10–3*, *StXTH18*, *StXTH19*, *StXTH20* and *StXTH23–1*) were not sensitive to touch treatment (Figures 1C, 3). The expression level of other *StXTH*s showed significant decrease.

## 4. Discussion and Conclusions

Plants are lifelong exposed to environmental mechanical factor, like rainfall, wind or touch. Plants are able to perceive and respond to these stimuli, via influencing plant growth, development thereby affecting their survival, as well as their evolutionary success.^2,9,10^ Responses of plants to these cues can be short term, like Ca^2+^-dependent signaling or molecular changes^14,33,34^ and as a consequence of those, long term, as well, like changes in growth and development.^8^ The three environmental cues, which transcriptional effects on the *XTH*s of potato and tomato were investigated in the present study, are very common during the life of plants. They can be both beneficial and harmful but definitely affect the metabolism, growth and development of plants. Even if rainfall is one of the sources of essential, life-giving water, it also carries potential dangers for the plants via possible transport and spread of pathogens, or parasites.^12,35^ It should be mentioned as a limitation of the study examining the effects of rain that its effect is a combined effect due to the nature of rain. Since the plants are not only mechanically affected by raindrops falling on them but the rainwater falling on the plants and reaching the soil from there may also affect the plants. Wind also affects the structure, morphology and growth of plants and triggers functional and developmental responses.^12,36^ Touch stimuli can be originated from a wide range of environmental components, such as from animals, neighboring plants, humans or abiotic surroundings, and can similarly elicit growth-developmental responses.^12,13,37^

Considering the fundamental roles of xyloglucan endotransglucosylases/hydrolases in cell growth,^15–18^ it should not been surprising if the gene expression of at least some of them were sensitive to environmental mechanical factors. Transcriptional responses were described for some *XTH* genes in *Arabidopsis* (in response to touch and sound),^11,14^ cucumber (in response to touch or injury)^19^ and potato (in response to ultrasound).^20^ However, to the best of our knowledge no other studies have been reported before that have addressed either the mechano-sensitivity of tomato *XTH* genes (*SlXTH*s) or mechano-inducible/suppressive properties of potato *XTH* genes (*StXTH*s), beyond our earlier study on the ultrasound-responsive expression of 11 potato *StXTH* genes.^20^

The expression of all *SlXTH*s was proven to be mechano-sensitive by at least one of the mechanical forces (rainfall, wind, touch) we studied. All *SlXTH*s changed their expression level in response to rainfall treatment, while 72% and 64% of the *SlXTH*s responded to the wind and touch treatment, respectively. The expression level of *SlXTH*2, *SlXTH4, SlXTH9, SlXTH20* and *SlXTH24* could be altered only by the rainfall treatment, but they were insensitive to the wind and touch.

Eleven out of these 35 putative *StXTH*s were proven earlier^20^ to be responsive to ultrasound (US). The 91%, 49% and 66% of the *StXTH*s responded to the rain, wind and touch treatment, respectively. The expression level of *StXTH1*, *StXTH2*, *StXTH5–1*, *StXTH7–1* and *StXTH9–2* decreased at all the treatments similar to the US treatment.^20^ The *StXTH12* and *StXTH16* were induced in response to the US treatment, while were repressed at rainfall, wind and touch treatments (Table 2).

**Table 2.** Comparative table of mechano-sensitivity of different *SlXTH*s and *StXTH*s investigated. Red indicates when the expression level increased, blue indicates when it decreased at any time points. *SlXTH*s are based on Saladié et al.^21^, *StXTH*s are based on Hidvégi et al.^20^.

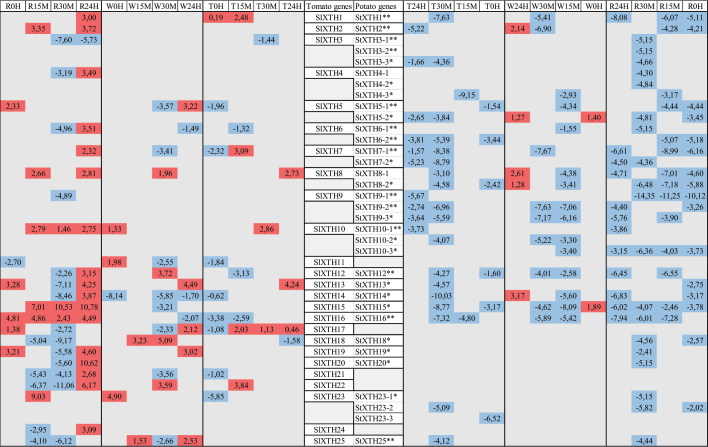

*.indicated as predictive XTH in NCBI database.

**.Response of different StXTHs to ultrasound treatment based on Hidvégi et al.^20^. W: wind, T: touch, R: rainfall treatment. 0 H, 15 M, 30 M and 24 H mean the time points at 0 hour, 15 min, 30 min and 24 hours, respectively.

The *SlXTH3* and *SlXTH4* similarly to *StXTH3–1*, *StXTH3–2*, *StXTH3–3*, *StXTH4–1* and *StXTH4–2* were not sensitive to wind treatment. The *SlXTH9* and *StXTH9–1* were not sensitive to rainfall and wind treatments. Neither *StXTH19* nor *SlXTH19* was sensitive to wind treatment. Similarly, neither *SlXTH20* nor *StXTH20* was sensitive to wind and touch treatments (Table 2).

Further investigations are required to discover how these mechano-sensitive *XTH*s can participate in the adaptive response to the environmental cues studied, in both species. Moreover, further searching for potential and presumably *XTH*s is necessary that may be induced mechanically, and further investigations are needed to discover how they can participate in the growth and developmental responses to environmental cues.
